# The Relationship between Portal Venous and Hepatic Arterial Blood Flow. I. Experimental Liver Transplantation

**DOI:** 10.1155/1996/90536

**Published:** 1996

**Authors:** F. Jakab, I. Sugár, Z. Ráth, P. Nágy, J. Faller

**Affiliations:** Department of Surgery Semmelweis University of Medicine & St. John Hospital Budapest Hungary

## Abstract

The relationship between the changes in portal venous and hepatic arterial blood flows, in the liver is a much disputed question, it has tremendous significance in the practice of transplantation, and an explanation has been available since 1981, when Lautt published the so-caled “adenosine washout theory”. According to our earlier observations the decrease of portal pressure or flow consistently led to an increase in hepatic artery flow. At the same time changes in hepatic artery flow or pressure seemed to produce only inconsistent effects on the portal circulation. In the present experiments liver transplantation (OLTX) was carried out on mongrel dogs by Starzl's method. Electromagnetic flow probes were placed on the hepatic artery and the portal vein before removal of recipient’s liver, and after completion of all vascular anastomoses to the newly inserted liver, during the recirculatory phase of OLTX. The flow probes were connected to a Hellige electromagnetic flowmeter, portal venous and systemic arterial pressures were also recorded.

The control HAF was 241±23 ml/min, the average PVF was 517±47 ml/min before removal of the recipients's liver. In the recirculatory phase the HAF increased, by 71±12% (p < 0.001). The PVF decreased in most animals after OLTX. The decrease was in average –40.2±3.5% (p < 0.001). The THBF calculated by adding the HAF and PVF showed a small, but not significant decrease during recirculation.

The systemic arterial pressure decreased slightly and portal vein pressure rose in most animals after OLTX. There was a substantial increase in portal inflow resistance and prehepatic arteriolar resistance and a decrease in hepatic artery resistance. The decrease of PVF after OLTX can be explained by progressive fluid accumulation in the liver parenchyma and increased sinusoidal and portal inflow resistance. The prolonged and continuous increase in hepatic artery flow during the recirculatory phase of OLTX may be due to the decrease of portal flow. The exact mechanism, by which a change in portal flow leads to arteriolar dilatation, can be most probably explained by the “adenosine washout theory” of Lautt.


*HPB Surgery*, 1996, Vol.10, pp.21–26
Reprints available directly from the publisher
Photocopying permitted by license only

© 1996 OPA (Overseas Publishers Association)
Amsterdam B.V. Published in The Netherlands
by Harwood Academic Publishers GmbH
Printed in Malaysia

### The Relationship between Portal Venous and Hepatic Arterial Blood Flow. I. Experimental Liver Transplantation

F. JAKAB, I. SUGÁR, Z. RÁTH, P. NÁGY and J. FALLER

Department of Surgery, Semmelweis University of Medicine & St. John Hospital. Budapest, Hungary.
Supported by a grant from Ministry of Welfare Hungary M-005/1990

*(Received 18 February 1994)*

The relationship between the changes in portal venous and hepatic arterial blood flows, in the liver is a much disputed question, it has tremendous significance in the practice of transplantation, and an explanation has been available since 1981, when Lautt published the so-caled "adenosine washout theory". According to our earlier observations the decrease of portal pressure or flow consistently led to an increase in hepatic artery flow. At the same time changes in hepatic artery flow or pressure seemed to produce only inconsistent effects on the portal circulation. In the present experiments liver transplantation (OLTX) was carried out on mongrel dogs by Starzl's method. Electromagnetic flow probes were placed on the hepatic artery and the portal vein before removal of recipient's liver, and after completion of all vascular anastomoses to the newly inserted liver, during the recirculatory phase of OLTX. The flow probes were connected to a Hellige electromagnetic flowmeter, portal venous and systemic arterial pressures were also recorded.

The control HAF was 241±23 ml/min, the average PVF was 517±47 ml/min before removal of the recipients's liver. In the recirculatory phase the HAF increased, by 71±12% ($p < 0.001$). The PVF decreased in most animals after OLTX. The decrease was in average –40.2±3.5% ($p < 0.001$). The THBF calculated by adding the HAF and PVF showed a small, but not significant decrease during recirculation.

The systemic arterial pressure decreased slightly and portal vein pressure rose in most animals after OLTX. There was a substantial increase in portal inflow resistance and prehepatic arteriolar resistance and a decrease in hepatic artery resistance. The decrease of PVF after OLTX can be explained by progressive fluid accumulation in the liver parenchyma and increased sinusoidal and portal inflow resistance. The prolonged and continuous increase in hepatic artery flow during the recirculatory phase of OLTX may be due to the decrease of portal flow. The exact mechanism, by which a change in portal flow leads to arteriolar dilatation, can be most probably explained by the "adenosine washout theory" of Lautt.

KEY WORDS: Afferent flow to the liver   interaction between portal venous and hepatic arterial blood flow   liver transplantation   recirculatory phase

### INTRODUCTION

The relationship between changes in portal venous and hepatic arterial blood flows, has tremendous significance in the practice of transplantation [5,9,18]. According to our earlier experimental observations a decrease of portal pressure or flow consistently led to an increase in hepatic artery flow [15]. At the same time changes in hepatic artery flow or pressure seemed to produce only inconsistent effects on the portal circulation[16].

The hemodynamics during liver transplantation can be examined, measured and evaluated only with great difficulties. Most patients undergoing liver transplantation have had long-standing portal hypertension with reduced PVF to the liver [4,5] and in patients with endstage liver disease there is high cardiac output (CO) and low total systemic vascular resistance[1–6].

Transplantation replaces the cirrhotic liver with a completely denervated liver, this reduces the portal

*Correspondence to:* Ferenc Jakab M.D.,Ph.D. Professor of Surgery Department of Surgery, Budapest, Uzoski Street 2p. Hungary, Diosàrok u. 1. Zip code: H–1145 Fax/Phone: 36-1 156-3049

pressure, and correction of the obvious pathological abnormality can be expected.

Data from the literature shows that transplantation does not restore the hepatic blood flow immediately after OLTX. (1–3,5,6).

In some flow-measurement studies on humans with OLTX the HAF to PVF ratios have been normal [7,18]. Payen [13] pointed out increased HAF in 7 out of 10 patients during portal vein clamping shortly after OLTX. Similar data are shown by Henderson [5].

The regulation of afferent hepatic bloodflow primarily depends on PVF, and the HAF changes reciprocal to PVF, –and the whole regulation mechanisms can be explained by the "adenosine washout" [9,10].

The aims of this experimental study were (a), to measure HAF and PVF during the reciculatory phase of OLTX in dogs (b), to evaluate and analyse the interaction between HAF and PVF.

### MATERIAL AND METHOD

Liver transplantations were carried out on mongrel dogs by Starzl's method using veno-venous bypass [8]. Electromagnetic flow probes were placed on the hepatic artery and the portal vein before removal of recipient liver, and then again after completion of all vascular anastomoses of the newly inserted liver, in the recirculatory phase of OLTX. The flow probes were connected to a Hellinge electromagnetic flowmeter, portal venous and systemic arterial pressures were recorded also. The results were calculated in terms of the mean ± standard error. Statistical significance of the changes was assessed with Student's "*t*" test applied to the percent differences between the control and the observed values.

### RESULTS

The control hepatic artery flow (HAF) was 241±23 ml/min (N = 14) the average portal vein flow (PVF) was 517±47 ml/min before removal of the recipient's liver. (Figure 1.) In the recirculatory phase the HAF increased to 414±39 ml/min, by 71±12% ($p < 0.001$). The PVF decreased was on average –40.2±3.5% ($p < 0.001$). The total hepatic in-flow (THBF) calculated by adding the HAF and PVF showed a small, but not significant decrease during recirculation. The control THBF was 758±50 ml/min or 39.0±3.1 ml/min/kg and in the recirculatory phase 754±48 ml/min. The systemic arterial pressure slightly decreased (Figure 2.), portal vein pressure rose in most animals after. OLTX (Figure 3). A substantial increase in total portal resistance could be observed, on the other hand arterial resistance diminished by 45% and portal vein resistance was more than doubled. The splanchnic arteriolar resistance increased significantly ($p < 0.05$).

Significant correlation was found between the increase of portal venous pressure and the changes in arterial and portal vein flows. (r HA = 0.6, $p < 0.01$; r pV = 0.69; $p < 0.01$). When the average HAF is plotted against the average portal vein flow it can be seen that the relationship is complex. With declining portal flow, arterial flow rises at first steeply, attaining a maximum above which there is a little change. For the first practically linear part of the regression curve the value of correlation coefficient was high ($r = 0.991$, $p < 0.01$) (Figure 4).

Regarding the relationship of percentage changes of PVF to HAF, the resulting curve is of the saturation type, i.e. with the decrease of portal vein flow HAF at first rises nearly in a linear fashion, attaining a maximum after which it does not change (Figure 5). In plotting the percentage changes of HAF against PVF a hyperbolic regression curve was obtained. (Figure 6).

In the 4 sham operated animals, the measured parameters practically did not change during the same observation period.

The decrease of PVF after OLTX can be explained by a progressive fluid accumulation in the liver parenchyma and increased sinusoidal and portal inflow resistance. The prolonged and continuous increase of HAF in the recirculatory phase of OLTX may be due to the decrease in portal flow. The exact mechanism, by which a change in portal flow leads to arteriolar dilatation can be explained by the "adenosine washout theory" of Lautt [9,10]. The interaction of the two afferent circulations in the liver has importance in OLTX, as that OLTX causes complete denervation of the liver. The interaction in liver hemodynamics with the priority of PVF was demonstrated in humans not only intraoperatively, but up to 7 days later by implanted Doppler probes [7,13].

It is likely that our observations are limited only to the recirculatory phase of OLTX, and the circulatory changes return to normal when the fluid accumulation and edema disappear from the liver. In human OLTX

Abbreviations:
Hepatic Artery Flow: HAF
Portal Venous Flow: PVF
Total Hepatic Blood Flow:THBF
Liver Transplantation: OLTX

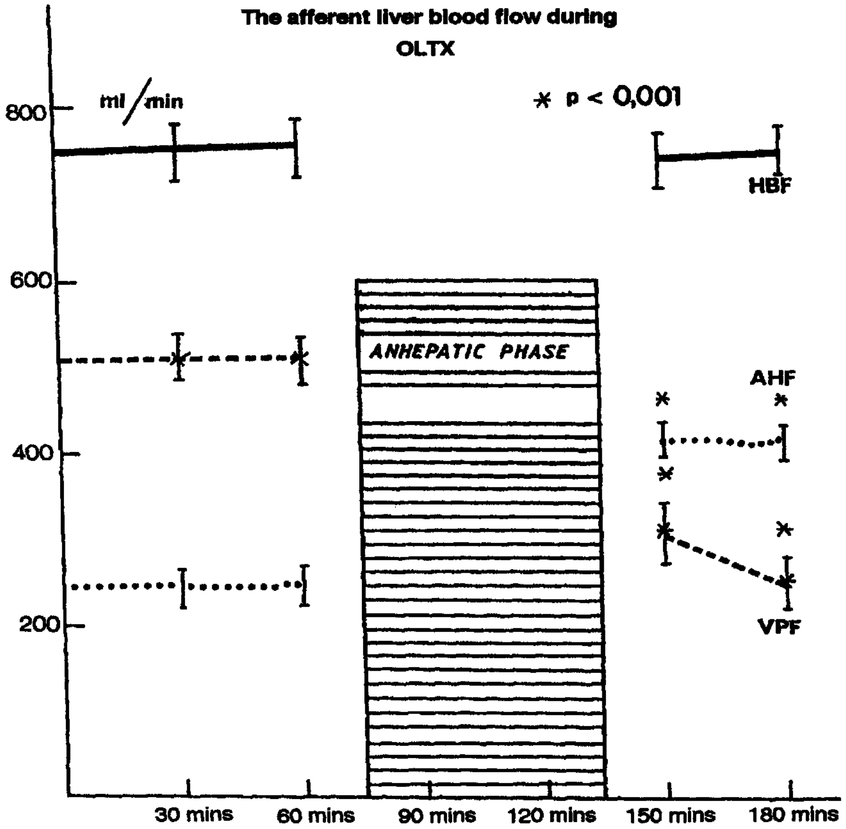

**Figure 1** The afferent circulation of liver during OLTX

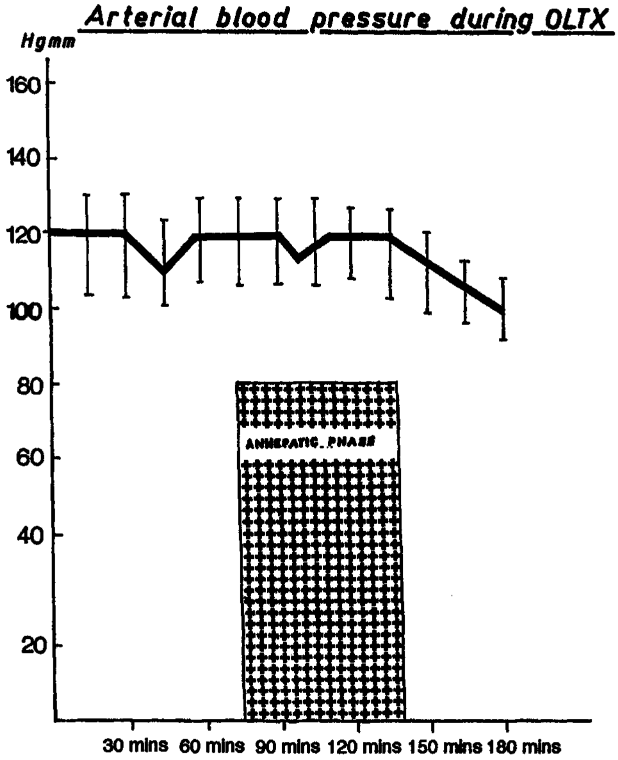

**Figure 2** Arterial blood pressure during OLTX.

analysis has shown a significant correlation between cardiac output and portal venous flow and a trend toward negative correlation between cardiac output and hepatic arterial flow. These data show that increased flow in a newly transplanted liver is predominantly portal venous flow and is associated with light cardiac output and reduced hepatic arterial flow [1–6]. These observations are not inconsistant with our resutls related to the interactions of the two afferent circulations during the recirculatory phase of OLTX.

The use of veno-venous bypass during the anhepatic phase of OLTX prevents the major disturbances in hemodynamics compared to OLTX done without veno-venous bypass.[8,14]

What are the implications of these data from experimental liver transplantation? The intraoperative measurements of the circulation to the completely denervated, transplanted liver gives evidence for the interaction of HA and PV system, and suggests a mechanism for the regulation of hepatic blood flow. The experimental model excludes many variables, which have to be taken into consideration in patients with end-stage liver disease. This study concludes, that the circulatory changes are only limited to the recir-

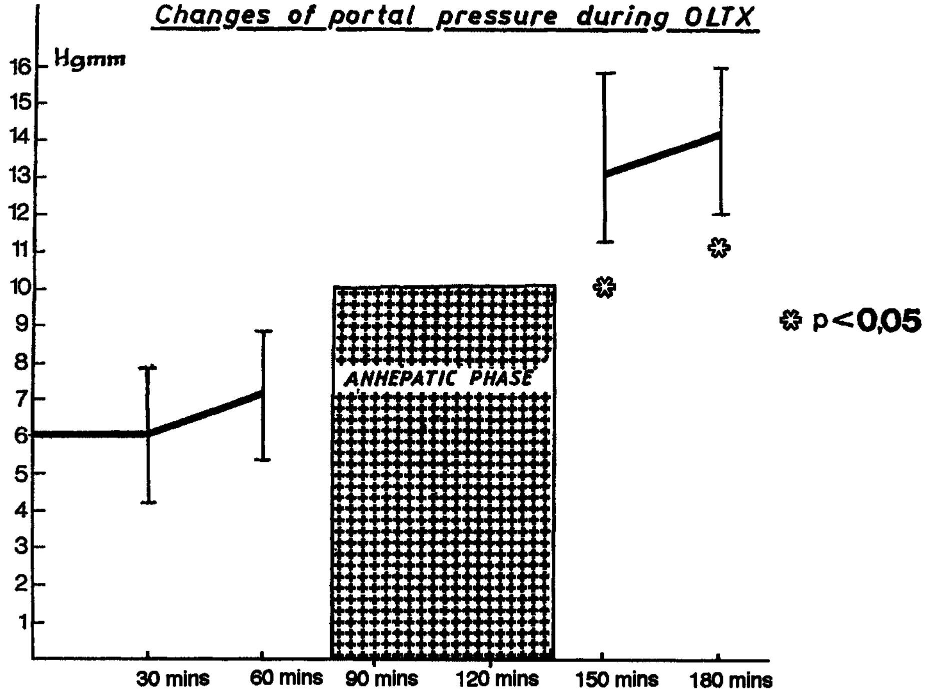

**Figure 3** Changes of portal pressure during OLTX

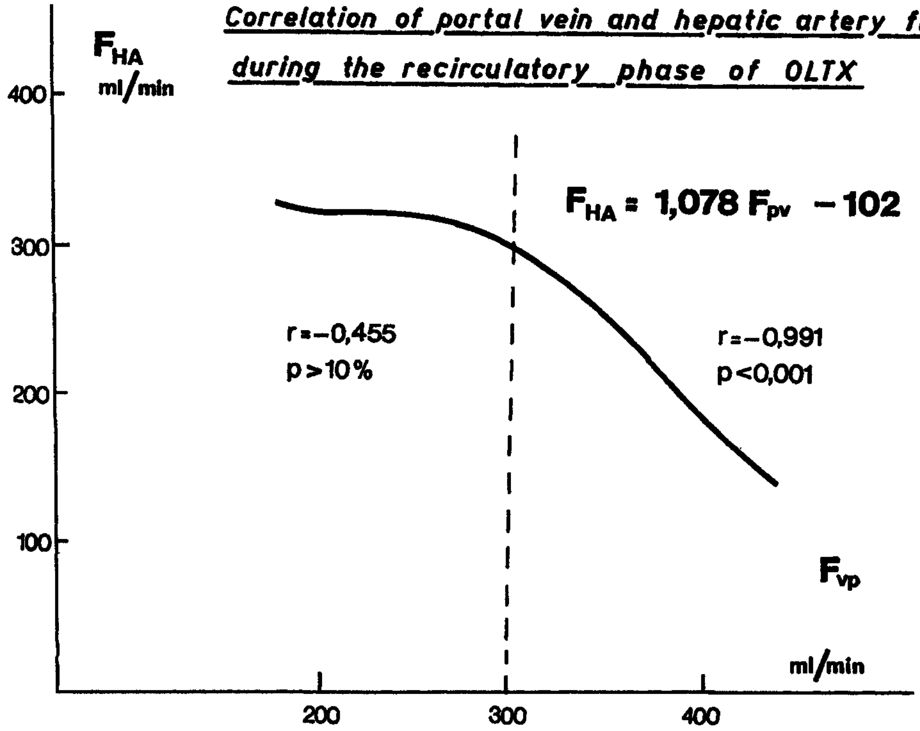

**Figure 4** Correlation of portal vein and hepatic artery flow during the recirculatory phase of OLTX

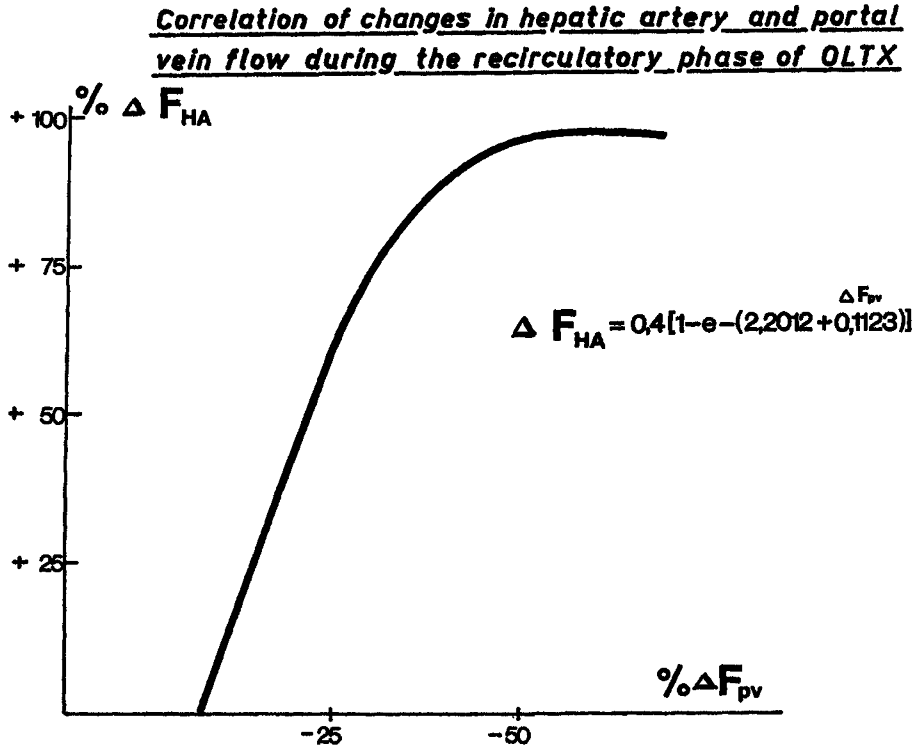

Figure 5 Correlation of changes in hepatic artery and portal vein flow during the recirculatory phase of OLTX

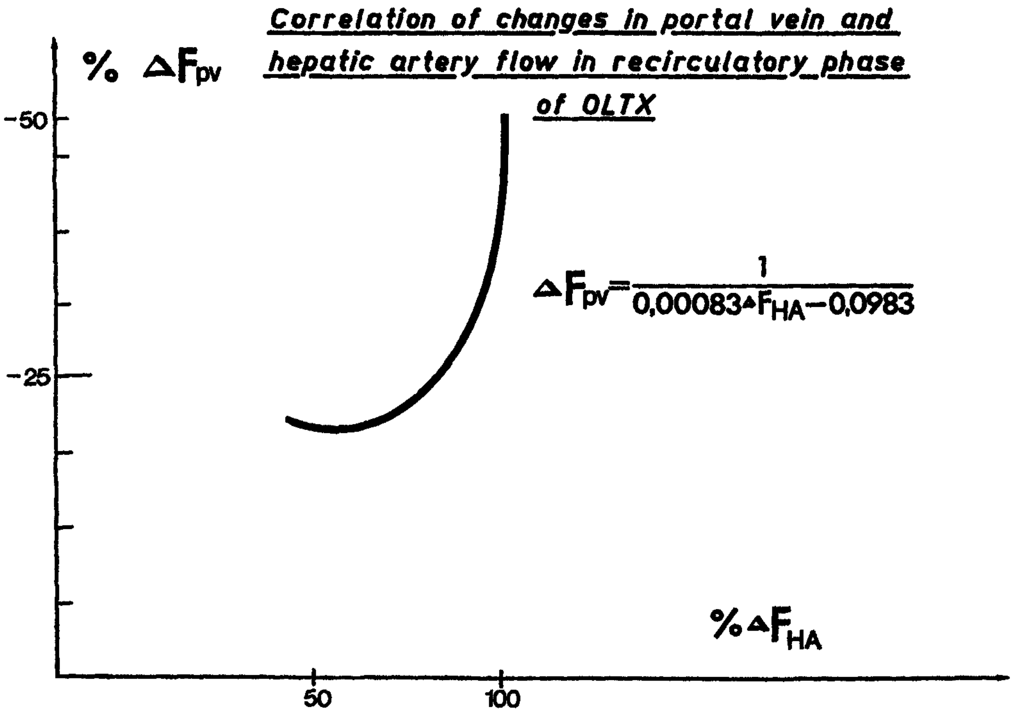

Figure 6 Correlation of changes in portal vein and hepatic artery flow in recirculatory phase of OLTX

culatory phase, when fluid accumulation and sinusoidal resistance has an effect on circulation. Later on, it is likely, that these circulatory changes revert to physiological control as shown by Textor[17] four weeks after liver transplantation in humans.

### REFERENCES

1. Hadengue A, Moreau R, Sogni P, *et al.* (1991) High cardiac Output After Liver Transplantation Is Due To Persistent Portosystemic Colleteral Blood Flow And Elevated Hepatic *Blood Flow Hepatology* 14. 57A.
2. Hadengue A. Lebrec D. Moreau R. *et a*l. (1993) Persistence of Systemic And Splanchnic Hyperkinetic Circulation In *Liver Transplant Patients Hepatology* **17:** 175–179.
3. Henderson J.M. Millikan W.J. Hooks M.H. *et al.* (1989) Increased Galactose Clearance After Liver Transplantation: A Measure Of Increased Blood Flow Through The Denervated *Liver Hepatology* **10:** 288–291.
4. Henderson J.M. Mackay G.J. Hooks M.H. *et al.* (1992) High Cardiac Output Of Advanced Liver Disease Persists After Orthotopic Liver Transplantation *Hepatology* **15:** 258–291.
5. Henderson J.M. Gilmore G.T. Mackay G.J. *et al.* (1992) Hemodynamics During Liver Transplantation: The Interaction Between Cardiac Output and Portal Venous and Hepatic Arterial Flows *Hepatology* **16:** 715–718.
6. Henderson J.M. (1992) Abnormal Splanchnic And Systemic Hemodynamics Of End-Stage Liver Disease. What Happens After Liver Transplantation? *Hepatology* **17:** 514–516.
7. Housin D. Fratacci M. Dupuy P. *et al.* (1989) One Week Of Monitoring Portal, Hepatic Arterial Blood Flow After Liver Transplantation Using Implantable Pulsed Doppler Microprobes *Transplant. Proc.* **21:** 2277–2278.
8. Kam I. Lynch S. Todo S. Dewolf A. McSteen F. Jakab F. Th.E. Starzl (1986) Low Flow Veno-Venous Bypass In Small animals and Pediatric Patients Undergoing Liver Replacement *Surgery, gynaecology, obstetrics* **33:** 163–167.
9. Lautt W.W. (1981) Role And Control Of The Hepatic Arteryn: Hepatic Circulation In Health And Disease. Edited By Lautt W.W. New York. Raven p. 203–220.
10. Lautt W.W. (1985) Mechanism And Role Of Intrinsic Regulation Of Hepatic Artery Blood Flow: Hepatic Arterial Buffer Response *Am. J. Physiol.* **249:** 549–556.
11. Navasa M. Feu F. Bosch J. *et al.* (1991) Systemic, Splanchnic And Humoral Changes After Orthotopic Liver Transplantation *J. Hepatol.* **13:** 455.
12. Navasa M. Feu F. Garcia J.C. *et al.* (1993) Hemodynamic And Humoral Changes After Liver Transplantation In Patients With Cirrhosis *Hepatology* **17:** 355–360.
13. Payen D.M. Fratacci M.D. Dupay P. *et al.* (1990) Portal and Hepatic Arterial Blood Flow Measurements of Human Transplanted Liver by Implanted Dopper Probes: Interest for Early Complications and Nutrition. *Surgery* **107:** 417–427.
14. Starzl T.E. Kaupp H.A. Borck D.R. *et al.* (1960) Reconstructive Problems in Canine Liver Homotransplantation with Special Reference to the Postoperative Role of Hepatic Venous Flow. *Surg. Gyn. Obstct.* **111:** 733-743.
15. G. Szabó, F. Jakab, Z. Magyar (1974) The Effect of Acute Cholestasis on Hepatic Circulation *Acta Med. Acad. Sci. Hung.* **31:** 229–239.
16. G. Szabó, F. Jakab, Z. Magyar (1974) The Mechanismus of the Effect of Increased Biliary Pressure on Hepatic Circulation *Acta Med. Acad. Sci. Hung* .**31:** 241–250.
17. Textor S.C. Wiesner R.H. Wisson D.J. (1993) Reversal Of Systemic Vasodilatation And Hyperdynamic Cardiac Output During Four Weeks After Liver Transplantation *Hepatology* **16:** 290A.
18. Yanaga K. Makowka L. Shimada M. Tzakis A. Starzl T.E. (1989) Hepatic Artery Thrombosis Following Pediatric Liver Transplantation: Assessment Of Blood Flow Measurements In Allografts *Clin. Transpl.* **3:** 184–189.

